# Child protection contact among children of culturally and linguistically diverse backgrounds: A South Australian linked data study

**DOI:** 10.1111/jpc.16364

**Published:** 2023-02-06

**Authors:** Razlyn Abdul Rahim, Rhiannon Pilkington, Alexandra M Procter, Alicia Montgomerie, Murthy N Mittinty, Katina D'Onise, John Lynch

**Affiliations:** ^1^ School of Public Health The University of Adelaide Adelaide South Australia Australia; ^2^ Robinson Research Institute The University of Adelaide Adelaide South Australia Australia; ^3^ Prevention and Population Health Wellbeing SA Adelaide South Australia Australia; ^4^ Bristol Medical School, Population Health Sciences University of Bristol Bristol UK

**Keywords:** child abuse and neglect, child maltreatment, culturally and linguistically diverse

## Abstract

**Aim:**

To describe the cumulative incidence of child protection (CP) system contact, maltreatment type, source of reports to age 7 years, and socio‐demographic characteristics for culturally and linguistically diverse (CALD) Australian children.

**Methods:**

We used CP, education, health, and birth registrations data for children followed from birth up to age 7 from the South Australian Better Evidence, Better Outcomes, Linked Data (SA BEBOLD) platform. Participants: SA born children enrolled in their first year of school from 2009 to 2015 (*n =* 76 563). CALD defined as non‐Aboriginal or Torres Strait Islander, spoken language other than English, Indigenous or Sign, or had at least one parent born in a non‐English speaking country. Outcomes measures: For CALD and non‐CALD children, we estimated the cumulative incidence (risk) of CP contacts up to age 7, relative risk and risk differences for all levels of CP contact from notification to out‐of‐home care (OOHC), primary maltreatment type, reporter type, and socio‐economic characteristics. Sensitivity analyses explored different population selection criteria and CALD definitions.

**Results:**

By age 7, 11.2% of CALD children had ‘screened‐in’ notifications compared to 18.8% of non‐CALD (risk difference [RD] 7.6 percentage points (95% confidence interval: 6.9–8.3)), and 0.6% of CALD children experienced OOHC compared to 2.2% of non‐CALD (RD 1.6 percentage points (95% confidence interval: 1.3–1.8)). Emotional abuse was the most common substantiated maltreatment type for CALD and neglect for non‐CALD. Among both groups, the most common reporter sources were police and education sector. Socio‐economic characteristics were broadly similar. Sensitivity analyses results were consistent with primary analyses.

**Conclusion:**

By age 7, CALD children had lower risk of contact with all levels of CP. Estimates based on primary and sensitivity analyses suggested CALD children were 5–9 percentage points less likely to have a report screened‐in, and from 1.0 to 1.7 percentage points less likely to have experienced OOHC.

## What is already known on this topic

1. In Australia, Aboriginal and/or Torres Strait Islander children are over represented in the child protection system.
2. In England and the United States, child protection contacts vary between ethnicities with over‐representation of Black and African American, and under‐representation of Asian children.
3. Disparities in child protection contact between ethnicities have been attributed to greater socio‐economic disadvantage.

## What this paper adds

1. Culturally and linguistically diverse (CALD) children were less likely to have contact across all levels of child protection from reporting to out‐of‐home care. By age 7, 11.2% of CALD children were screened‐in compared to 18.8% of non‐CALD.
2. Sensitivity analysis suggested CALD children were 5–9 percentage points less likely to have a report screened‐in, and from 1.0 to 1.7 percentage points less likely to have experienced out‐of‐home care.
3. CALD and non‐CALD groups differed by the type of maltreatment, but were similar by source of report, or on background socio‐economic factors.

Child maltreatment is abuse and/or neglect of a child, and has long been recognised as an important public health issue.^1^, ^2^, ^3^ In 2020–2021, child protection (CP) agencies in Australia received 531 900 notifications for 293 600 children.^4^ In South Australia (SA), 25% of children were notified at least once to CP services by the age of 10 years.^5^ In New South Wales (NSW), 13.8% of children are the subject of screened‐in notifications by age 5.^6^ Adverse effects of maltreatment on mental^7^, ^8^ and physical health, education attainment, labour market participation,^9^ and increased risk of antisocial behaviours^10^, ^11^ have been shown in previous research.

Racial or ethnic disparities in CP contact are well recognised.^12^ In the United States,^13^, ^14^, ^15^ Canada,^16^ Australia^17^, ^18^ and New Zealand,^19^ children of First Nations communities are over‐represented in the CP system, as a consequence of colonialism, dispossession and systemic discrimination. The CP experience is heterogeneous within subgroups of racialised or ethnic minority groups. In England, the rate of children substantiated was higher in Mixed heritage (84 per 10 000) compared to White (50 per 10 000) children.^20^ Asian children had a lower rate compared to White at 23 per 10 000 children.^20^ However, the rate of Black (87 per 10 000) and mixed heritage (99 per 10 000) children in out‐of‐home care (OOHC) far exceeded that of White children (64 per 10 000).^20^ In the United States, approximately 20% of African American children and between 10 and 13% of Latino/Hispanic children were substantiated for maltreatment compared to 10% of White children.^13^, ^14^ Conversely the cumulative incidence of substantiated maltreatment in Asian and Pacific Islander children was less than 5%.^13^, ^14^ In both countries the disparities in contact patterns between White and racialised or ethnic minority children have been attributed to greater socio‐economic disadvantage.^12^, ^13^, ^14^, ^20^, ^21^, ^22^, ^23^, ^24^, ^25^ While the over‐representation of Aboriginal and Torres Strait Islander children and families in the CP system has long been documented, little is known about the CP contact of children from other racialised non‐Indigenous ethnic minority groups in Australia.

Culturally and linguistically diverse (CALD) background is a term unique to Australia. It is intended to identify groups who are not of the mainstream English‐speaking Anglo‐Celtic group and do not identify as Aboriginal or Torres Strait Islander (Australia's First Nations population). Although the term CALD is widely used in public and policy discourse, there are no consensus guidelines on how to enumerate this heterogeneous population.^26^ Despite the availability of data collection frameworks for being counted as CALD, such as country of birth and language spoken, enumerating the CALD population lacks an operational definition.^26^, ^27^ Using data from the 2016 Australian Census, we have shown that applying different definitions that might identify the CALD population results in an estimated proportion of CALD children aged 0–17 ranging from 11 to 44%.^27^

Only five previous studies have discussed CP contact for CALD children in Australia. Three of these involved considerations of the need to understand cultural diversity in regard to CALD and refugee CP contact but provided no data on incidence or prevalence.^28^, ^29^, ^30^ These studies explored experiences of children within the CP system. Two studies conducted case file reviews with sample sizes of 81 and 120.^29^, ^30^ One surveyed organisations providing 20% of OOHC services in Victoria.^28^ Hotton and Raman^31^ presented data on country of origin among 279 children referred for child maltreatment assessment in South West Sydney health district, noting large variation among CALD groups and higher representation of Pacific Island children, but this study did not have whole population denominators to determine incidence or prevalence.

Our study objective is to describe,^32^, ^33^ cumulative incidence (risk) of child maltreatment, maltreatment type, and source of notification up to age 7, as well as socio‐demographic characteristics using our preferred definition of CALD and non‐CALD children attending government schools in South Australia (SA). Sensitivity analyses explored different population selection criteria and CALD definition, and accounted for the over‐representation of Aboriginal and/or Torres Strait Islander children in the CP system. This study utilised administrative data of all reports to the statutory CP agency. These reports are one indicator of maltreatment, of which the true burden in the community is unknown.

## Methods

### Data sources

This study used whole population de‐identified data from the SA Better Evidence, Better Outcomes Linked Data platform. Child protection records were linked with data from the Perinatal Statistics Collection, Births, Deaths and Marriages Registry, Government School Enrolment Census (SEC) and the 2009, 2012 and 2015 Australian Early Development Census (AEDC). Linkage was conducted by SANT Datalink, an independent agency.^34^ The estimated false positive linkage error is 0.4%.^35^

### Population

**Fig. 1:**
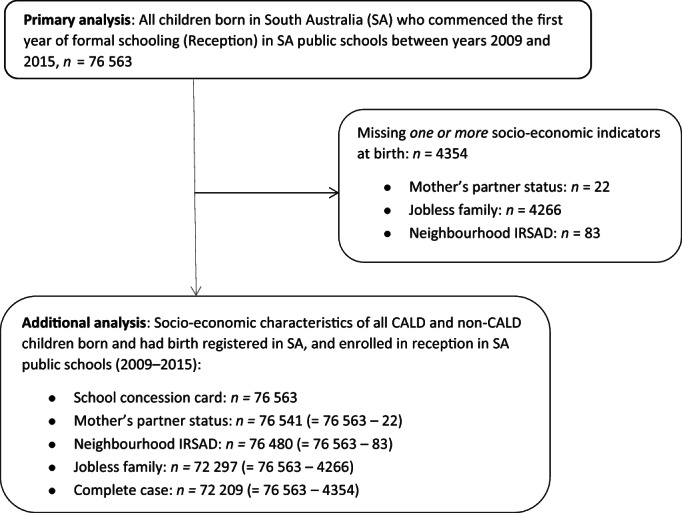
Flowchart of study population and sample for primary and additional analysis. CALD, culturally and linguistically diverse; IRSAD, Index of Relative Socioeconomic Advantage and Disadvantage.

Primary analysis included children born in SA who started in a government school between 2009 and 2015 (*n* = 76 563), corresponding to children born 2003–2010. Additional analysis describing socio‐economic characteristics was limited to children with complete information recorded at birth (*n* = 72 209) (Fig. 1).

### Child protection contact

CP data cover the entire CP system from initial notifications by mandatory and non‐mandatory sources to experience of OOHC. These include initial notifications, notifications not screened‐in, screened‐in notifications not investigated, investigations not substantiated, substantiations, and OOHC. Other CP data included child age, reporter source (e.g. health, education, police), primary alleged (screened‐in notifications) and substantiated maltreatment type as determined by the Department for Child Protection. Definitions of maltreatment type are included in the Supporting Information.

### CALD identification

Consistent with the theory behind understanding social experiences of racialised groups such as CALD,^36^, ^37^ we used an indicator of CALD based on a combination of language(s) spoken at home and parental country of birth. As many CALD children born in Australia have parents who were immigrants, the child's country of birth was not used. We defined CALD children as non‐Aboriginal or Torres Strait Islander who spoke a first or second language other than English, Celtic, Australian Indigenous or sign at home, and/or had at least one parent born in countries other than Australia, New Zealand, United Kingdom, Ireland, United States and Canada. These items were only available in the SEC for children attending government schools. Non‐CALD were all Aboriginal or Torres Strait Islander and non‐Indigenous peoples who spoke English, Celtic, any Australian Indigenous or sign language at home and had both parents born in Australia, New Zealand, United Kingdom, Ireland, United States, Canada or South Africa.

For sensitivity analysis to include children in non‐government schools, we used data from the AEDC to define CALD. This is similar to the definition above except that the child's country of birth was used, as parental country of birth was not available. Those from a non‐English language background and/or born in non‐English speaking countries were classified as CALD.

### Socio‐economic disadvantage

Socio‐economic indicators were obtained from perinatal and birth registration data, and SEC. This included mother's partner status, mother's residential postcode, and father's and mother's workforce participation. Jobless families were those with no parent(s) participating in the workforce.^38^ The Index of Relative Socioeconomic Advantage and Disadvantage was based on residential postcode, and a school concession card that subsidises education expenses.^39^

### Analysis

#### Primary analysis

Cumulative incidence of all levels of contact with CP was calculated at each age up to 7 years, by dividing the number of CALD and non‐CALD children who had contact with each level of CP by the total number of children. We estimated the risk of ever having CP contact by age 4 (to capture experiences before school), and by age 7, and relative risks and differences. Within the population of children who were reported we: (i) calculated proportions of children by age at first notification in infancy (<1 year), early childhood (1–4 years) and the first 3 years of formal schooling (5–7 years), by sex; (ii) calculated reporter type for reports up to age 4, and between ages 5 and 7; and (iii) examined primary alleged and substantiated maltreatment type for all reports up to age 7.

#### Additional analysis

We explored socio‐economic characteristics of families of CALD and non‐CALD children on a subset of the primary analysis population with complete data.

#### Sensitivity analysis

##### Agreement between two measures of CALD

We calculated classification agreement in a sub‐population with CALD indicators based on the SEC and AEDC. This population comprised children with an AEDC in government schools in 2009, 2012 or 2015, and included children not born in SA. To account for imbalanced outcomes we used the agreement coefficient (AC_1_) as a measure of concordance^40^, ^41^ between the measures of CALD (see Table S1, Supporting Information).

##### Variations to the study population

We undertook four sensitivity analyses: (i) We included children attending independent schools in addition to those in government schools, and used an alternative definition of CALD derived from the AEDC (where child's country of birth was used as parental country of birth was not available); (ii) We limited the analysis to an overlapping subset of the study population comprised of children who sat the AEDC in a government school in 2009, 2012 and 2015, and who were identified as CALD by either definition; (iii) Given the over‐representation of Aboriginal and Torres Strait Islander children in the CP system, the third sensitivity analysis excluded those children from the non‐CALD comparison group; and (iv) We repeated the primary, and the first and second sensitivity analyses to include all children, including migrant children who were not born in SA and so would not have a perinatal or birth record but could have AEDC and school records.

### Ethics approval

Ethics approval was granted by the Human Research Ethics Committee of the South Australian Department of Health and Ageing (SA Health) (ref HREC/13/SAH/106; HREC/13/SAH/106/AM18), the University of Adelaide Human Research Ethics Committee (H‐185‐2011), and the Aboriginal Health Council Research Ethics Committee (ref 04‐13‐538).

## Results

### Primary analysis

**Fig. 2:**
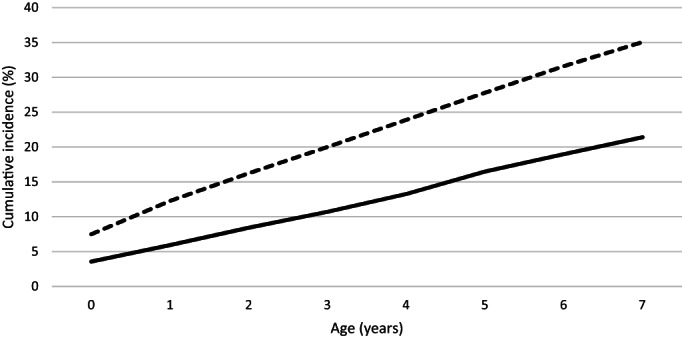
Cumulative incidence of child protection notifications up to age 7 and enrolled in school from 2009 to 2015, followed to the end of 2017 (*n* = 76 563). (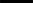), culturally and linguistically diverse (CALD); (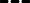), non‐CALD.

Of the 76 563 children in the study population, 12.1% were CALD. Figure 2 shows the cumulative incidence of at least one notification was lower in CALD children at all ages. The cumulative incidence of CP notifications was 3.6 and 7.5% at under age 1 for CALD and non‐CALD children respectively. By age 7, the cumulative incidence of CP notifications was 21.4% for CALD and 35.1% for non‐CALD children. This corresponds to risk differences of 3.9 percentage points at age 0, and 13.7 percentage points at age 7. Cumulative incidence up to age 7 by different birth cohorts is shown in Figure S1 (Supporting Information).

Table 1 shows all CP contact patterns. By ages 4 and 7, the risk of CP contact was lower for CALD compared to non‐CALD children across all levels of contact from notifications to OOHC. For example, by age 7, the risk difference between CALD and non‐CALD children for screened‐in was 7.6 percentage points, whilst for OOHC it was 1.6 percentage points. Risk ratios increased with greater intensity of contact so that by age 7 non‐CALD children had over three times the risk of being in OOHC (risk ratio = 3.52, 95% confidence interval (CI) 2.70–4.58).

**Table 1:** Child protection contact by ages 4 and 7 years for children born in SA and in SA public schools between 2009 and 2015, followed to the end of 2017 (*n* = 76 563)

Child protection contact	Non‐CALD, *n* = 67 339	CALD, *n* = 9224	Total, *n* = 76 563	Risk difference (95% CI) (no‐nCALD‐ CALD)	Risk ratio (95% CI) (non‐CALD/CALD)
	% with contact	% with contact	*n* (%)		
By age 4 (prior to school years)					
Notified	19.8	11.9	14 431 (18.8)	7.8 (7.1, 8.6)	1.66 (1.56, 1.75)
Screened in	14.6	8.0	10 577 (13.8)	6.6 (5.9, 7.2)	1.82 (1.69, 1.95)
Investigated	8.0	3.4	5693 (7.4)	4.6 (4.2, 5.0)	2.35 (2.11, 2.63)
Substantiated	4.1	1.5	2914 (3.8)	2.7 (2.4, 3.0)	2.82 (2.38, 3.35)
OOHC	1.6	0.5	1153 (1.5)	1.1 (0.9, 1.3)	3.09 (2.32, 4.11)
By age 7 (including first 3 years of school)					
Notified	26.4	17.9	19 465 (25.4)	8.5 (7.7, 9.4)	1.48 (1.41, 1.54)
Screened in	18.8	11.2	13 696 (17.9)	7.6 (6.9, 8.3)	1.68 (1.58, 1.78)
Investigated	9.9	4.2	7078 (9.2)	5.7 (5.3, 6.2)	2.36 (2.14, 2.61)
Substantiated	5.6	1.9	3931 (5.1)	3.7 (3.4, 4.0)	2.99 (2.57, 3.48)
OOHC	2.2	0.6	1522 (2.0)	1.6 (1.3, 1.8)	3.52 (2.70, 4.58)

CALD, culturally and linguistically diverse; CI, confidence interval; OOHC, out‐of‐home care.

Table 2 shows alleged and substantiated maltreatment type for screened‐in reports. Children may have multiple alleged and substantiated reports therefore may have more than one type of primary maltreatment. The maltreatment type at substantiation may differ from the alleged type following CP investigation. Emotional abuse was the most common alleged maltreatment, affecting 39.9% of CALD and 32.8% of non‐CALD children by age 7. Emotional abuse and neglect were the most common substantiated maltreatment for CALD and non‐CALD children, respectively, affecting approximately 40% of children in each group. CALD children were less likely to be screened‐ in or substantiated for neglect and sexual abuse compared to non‐CALD children. However, they are more likely to be screened in or substantiated for physical abuse than non‐CALD children. The proportion of CALD children who were substantiated for sexual abuse was approximately half that of non‐CALD children.

**Table 2:** Primary maltreatment type by screened‐in allegations for 13 696 children, and substantiations for 3931 children up to age 7 years†, followed to the end of year 2017

Maltreatment type	Screened in allegations (*n* children = 13 696)				Substantiations (*n* children = 3931)			
	Non‐CALD (*n* children = 12 664)		CALD (*n* children = 1032)		Non‐CALD (*n* children = 3759)		CALD (*n* children = 172)	
	Allegations, *n*	Column percentage (%)	Allegations, *n*	Column percentage (%)	Substantiations, *n*	Column percentage (%)	Substantiations, *n*	Column percentage (%)
Emotional abuse	7970	32.8	616	39.9	1848	35.9	85	41.9
Neglect	7219	29.7	352	22.8	2029	39.5	61	30.0
Physical abuse	5632	23.2	410	26.6	859	16.7	49	24.1
Sexual abuse	3477	14.3	166	10.7	406	7.9	8	3.9
Total screened in and substantiations	24 298	100	1544	100	5142	100	203	100

† Children can have more than one type of maltreatment due to multiple screened‐in or substantiated reports.

CALD, culturally and linguistically diverse.

Age patterns showed that of children notified to CP, 19.9% of CALD compared to 28.3% of non‐CALD children were notified before age 1, and 33.4% of CALD children were notified when school aged (5–7 years), compared with 25.2% of non‐CALD children. There were few sex differences with the exception of a higher proportion of CALD children substantiated by age 7 were females (55.2%) (age and sex data not shown).

**Fig. 3:**
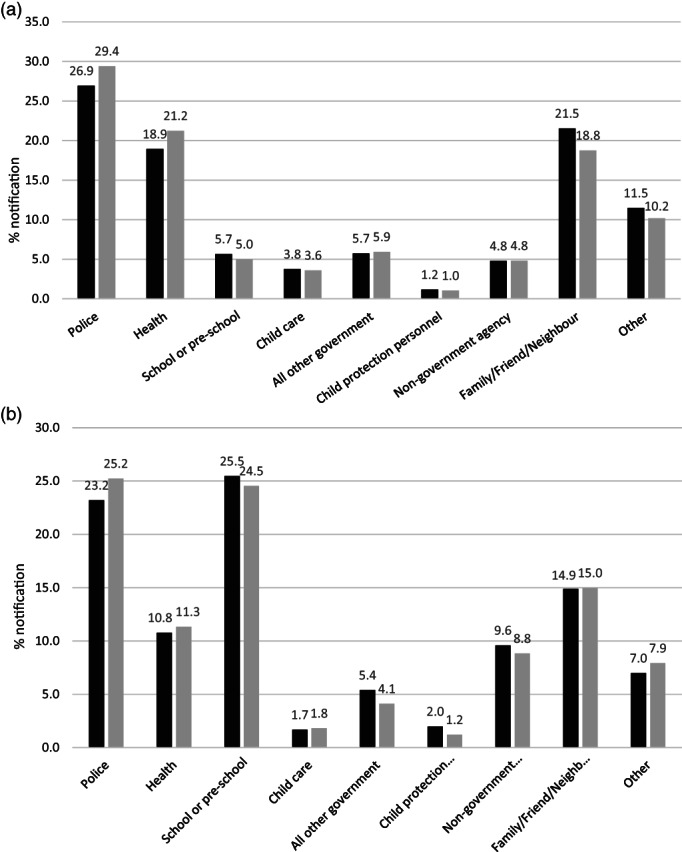
(a) Sources of all reports for children first notified by age 4 years. Denominator: number of notifications for children born in SA and attended reception in SA public schools between years 2009 and 2015 (*n* = 50 790). (b) Sources of all reports for children first reported between ages 5 and 7 years. Denominator: number of notifications for children born in SA and attended reception in SA public schools between years 2009 and 2015 (*n* = 40 968). Each child may be notified more than once. (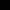), non‐culturally and linguistically diverse (CALD); (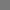), CALD.

Figure 3a shows the distribution of reporter type was similar with police as the main source under age 4, followed by reports from health, and family and personal social networks. Figure 3b shows that schools and police accounted for over half of reports in CALD and non CALD children aged 5 to 7 years.

### Socioeconomic characteristics

Table 3 shows few socio‐economic differences, except mothers of CALD children were more likely to be partnered at birth.

**Table 3:** Socio‐economic characteristics of culturally and linguistically diverse (CALD) and non‐CALD children

	Non‐CALD, *n* = 63 771	CALD, *n* = 8438	Total, *n* = 72 209
	Column percentage (%)	Column percentage (%)	*n* Column percentage (%)
Mother's partner status			
Partner	84.3	90.2	85.0
No partner	15.7	9.8	15.0
Jobless family			
No	89.6	86.1	89.2
Yes	10.4	13.9	10.8
IRSAD area‐based decile within Australia based on IRSAD for child year of birth			
Most disadvantaged	17.7	16.3	17.5
2	10.0	10.2	10.0
3	11.3	8.9	11.0
4	13.7	12.2	13.5
5	11.2	10.6	11.1
6	6.3	6.8	6.3
7	8.1	9.9	8.3
8	10.2	10.5	10.3
9	8.4	10.8	8.7
Most advantaged	3.1	3.8	3.2
School concession card			
No	80.7	80.6	80.7
Yes	19.3	19.4	19.3

Study population: complete cases of children born and had birth registered in SA who attend reception in a SA public school between years 2009 and 2015, followed up until the end of year 2017 (*n* = 72 209).

IRSAD, Index of Relative Socioeconomic Advantage and Disadvantage.

### Sensitivity analyses

Agreement between the two definitions of CALD (AC_1_ = 0.92) was high (see Table S1, Supporting Information).

Sensitivity analyses were consistent with the primary analysis where the risk of all forms of contact was lower in CALD children. Primary analysis showed that by age 7, the risk difference between CALD and non‐CALD was 7.6 percentage points. Sensitivity analyses bounded these risk differences from 5 to 9 percentage points. For OOHC risk differences ranged between 1.0 and 1.7 percentage points. Results of the sensitivity analyses are shown in Tables S2–S7 (Supporting Information).

## Discussion

Previous Australian studies exploring CP contact in CALD children sampled those known to the CP system therefore did not aim to quantify the incidence of contact in the broader community. This study is the first in Australia to examine the risk of CP contact for CALD children at population level.

Using whole‐of‐population linked administrative data we demonstrated that the risk of all forms of contact with the CP system up to age 7 was lower for CALD than non‐CALD children. Our primary analysis showed that by age 7, CALD children were 7.6 percentage points (95% CI: 6.9–8.3) less likely to have been screened‐in, and of 1.6 percentage points (95% CI: 1.3–1.8) less likely to have experienced OOHC than non‐CALD children. In the absence of self‐identification, there is no gold standard for defining CALD populations. Using different definitions of CALD applied in different, and the same populations showed consistent findings. The magnitude of risk differences depends on which definitions and populations are used, but our estimates and sensitivity analyses showed that CALD children were from 5 to 9 percentage points less likely to have reached a risk threshold to be screened‐in, and from 1.0 to 1.7 percentage points less likely to have experienced OOHC. CALD and non‐CALD families appeared to have broadly similar socio‐economic profiles. Although beyond the scope of this study, further research could investigate these patterns of CP contact. A proposed framework theorising ethnic inequalities in child welfare identified different approaches, including examining data artefacts such as poor identification of ethnicity, ‘demand’ factors like cultural practices in child rearing and population distribution of known risk factors for child maltreatment, and ‘supply’ factors such as systemic bias and varying opportunities for surveillance.^20^

We highlight some challenges to our study and how these were considered. First, the lack of an official definition of CALD poses a challenge in identifying this group. There is no consistency in how information to identify CALD are collected in administrative datasets used in this study. We endeavoured to address this issue by utilising a preferred definition which maximised the use of information consistent with the data collection framework for CALD specified by the Australian Bureau of Statistics. Additionally, we performed sensitivity analysis using an alternative definition of CALD which validated the findings of the primary analysis. Second, despite the obvious heterogeneity in a diverse CALD population, we did not do a further breakdown of CALD subgroups due to the challenge in creating meaningful subgroups of CALD. If CALD children were to be meaningfully categorised according to language spoken or parental country of birth, the small numbers that would be generated raises the risk of re‐identification. Conversely, creating broad categories (e.g. Asian, African) as has been done in studies elsewhere^20^, ^21^ may not support meaningful interpretation, and may inadvertently stigmatise subsets of the CALD community. Lastly, many CALD people would identify as immigrants, but we were not able to examine this because data on immigrant status is not currently available from the Australian government. Information on immigration status may enable further analysis to identify high need CALD subpopulations, such as refugees who are identified by visa subclass.

## Conclusion

Up to age 7, CALD children had lower risk of contact across all levels of the CP system in South Australia, from notification to OOHC. CALD and non‐CALD groups differed by maltreatment type, but were similar for source of notification, or on background socio‐economic factors. This general pattern of lower risk among CALD populations was consistent across sensitivity analyses exploring different definitions of CALD and using different study populations.

## Supporting information


**Fig. S1.** Cumulative incidence of child protection reports by age and year commenced Reception, 2009–2015 (A): CALD (B): non‐CALD.
**Table S1.** Number of children classified as CALD and non‐CALD by the preferred and alternative definitions of CALD (*n* = 34 217).
**Table S2.** Child protection contact by ages 4 and 7 years for children born in SA and sat the AEDC in SA in years 2009, 2012 and 2015, followed to the end of 2017 (*n* = 46 759).
**Table S3.** Child protection contact by ages 4 and 7 years for children born in SA and sat the AEDC in SA government school in years 2009, 2012 and 2015, followed to the end of 2017 (*n* = 29 172).
**Table S4.** Child protection contact by ages 4 and 7 years for non‐Indigenous children born in SA and attended reception in government schools between years 2009 and 2015, followed to the end of 2017 (*n* = 72 394).
**Table S5.** Child protection contact by ages 4 and 7 years for all children who attended reception in government schools between years 2009 and 2015, followed to the end of 2017 (*n* = 91 395).
**Table S6.** Child protection contact by ages 4 and 7 years for all children who sat the AEDC in SA in government and independent schools in years 2009, 2012 and 2015, followed to the end of 2017 (*n* = 55 232).
**Table S7.** Child protection contact by ages 4 and 7 years for all children who sat the AEDC in SA government schools in years 2009, 2012 and 2015, followed to the end of 2017 (*n* = 34 271).
